# Four Approaches for Implementing Interprofessional Education

**DOI:** 10.1093/geroni/igab046.400

**Published:** 2021-12-17

**Authors:** Jennifer Mendez, Jennifer Mendez

## Abstract

Using Microsoft Teams, the students from Psychology and marketing at Upper Iowa University, create a marketing plan focused on proposing a product or service targeting older adults. The Michigan LEND program, engages a minimum of 4 disciplines in practice online simulation approaches to respond to a case study. At Wayne State University and University of Detroit Mercy, during a zoom visit with community dwelling 50+ old adults, students from 9 disciplines collaborate on recommendations, referrals, and resources to improve health and/or quality of life. Marquette University students from 10 health professions participate in a series of four half-day workshops, designed in alignment with the Interprofessional Education Collaborative (IPEC) core competencies.

